# Changes in oxygen saturation and the retinal nerve fibre layer in patients with optic neuritis associated with multiple sclerosis in a 6‐month follow‐up

**DOI:** 10.1111/aos.14463

**Published:** 2020-05-12

**Authors:** Tereza Svrčinová, Pavel Hok, Irena Šínová, Tomáš Dorňák, Michal Král, Petra Hübnerová, Jan Mareš, Petr Kaňovský, Martin Šín

**Affiliations:** ^1^ Department of Neurology Faculty of Medicine and Dentistry University Hospital Palacký University Olomouc Olomouc Czech Republic; ^2^ Department of Ophthalmology Faculty of Medicine and Dentistry University Hospital Palacký University Olomouc Olomouc Czech Republic

**Keywords:** multiple sclerosis, optic neuritis, optical coherence tomography, oximetry

## Abstract

**Purpose:**

Optic neuritis (ON) is an inflammatory demyelinating disorder of the optic nerve, which can be the first manifestation of multiple sclerosis (MS). The main goal was to assess changes in the retinal nerve fibre layer (RNFL) and in retinal oxygen saturation [arterial (AS), venous (VS) and arterio‐venous (A‐V) difference] in the affected and unaffected eye.

**Methods:**

Fifty patients with ON due to MS within 3 months of onset of symptoms were enrolled (17 males, mean age 35.3). All patients were examined at baseline (V1) and after 6 months (V2) using optical coherence tomography (OCT) to get RNFL values; automatic retinal oximetry to obtain saturation values; and ultrasound to exclude arterial stenosis, and orbital colour Doppler imaging was performed in the ophthalmic artery.

**Results:**

At V1, AS was significantly increased in affected eye compared to unaffected eye (99.5% versus 98.0%, p = 0.03). Significant decrease in A‐V difference from baseline was detected in both eyes for ON eye: 32.0% versus 29.0%, p = 0.004; for fellow eye: 31.4% versus 30.0%, p = 0.04. We did not observe any changes in retinal vessel diameter. There were no changes observed in blood flow in ophthalmic artery. At V1, there were no significant differences in RNFL, and significant loss of RNFL was confirmed in the affected eye at V2 (95 *μ*m versus 86 *μ*m, p = 0.0002) and in comparison with the fellow eye (86 *μ*m versus 94 *μ*m, p = 0.0002). There were no correlations between RNFL and saturation values at V1, although at V2, there was a negative correlation between the RNFL and AS (Spearman's rho = −0.480, p = 0.003) and between the RNFL and VS (rho = −0.620, p = 0.00007).

**Conclusion:**

Retinal oximetry is altered in both eyes in MS patients with unilateral ON. During the course of the disease, the retinal oxygen consumption decreases to a different degree in each eye and this change is not completely followed by changes in the RNFL thickness, suggesting either sub‐clinical ON or systemic effects in the clinically unaffected eye. Since this is the first and initial longitudinal evaluation of the saturation changes in MS patients, the clinical value of these findings needs to be deeper evaluated in the future studies.

## Introduction

Multiple sclerosis (MS) is chronic immune‐mediated inflammatory disease with subsequent neurodegenerative processes; it typically affects younger people of working age and is also a major cause of young people's nontraumatic neurological disability in the United States and Europe (Dutta & Trapp 2011). Multiple sclerosis (MS) impacts social life (Rao et al. 1991; Stenager et al. 1994; Hakim et al. 2000) and represents financial burden for economy in the Western world (Kobelt et al. 2017; Hartung 2017). Multiple sclerosis (MS) patients benefit from early treatment, which reduces the risk of the next relapse and disability progression (Freedman et al. 2014). Because of that, early diagnosis and early treatment intervention to stop the progression of the disease are of paramount interest. Unfortunately, there is currently no reliable biomarker for monitoring the disease progression and response to treatment (Axisa & Hafler 2016). Understanding the pathophysiological processes of MS may, therefore, lead to improved treatment targeting with subsequent decrease of disability progression, improvement of quality of life and cost reduction.

One of the most common MS manifestations is optic neuritis (ON) (Britze & Frederiksen 2018) characterized by the destruction of the myelin sheath and nerve fibres in the optic nerve, resulting in impaired vision or blindness (Toosy et al. 2014). The diagnosis of ON is based on clinical signs and symptoms. Further diagnostic tools include magnetic resonance imaging, cerebrospinal fluid analysis and visual evoked potentials (VEP) that have been long used to detect well‐described characteristic changes in MS patients (Halliday et al. 1973; Beck et al. 1993; Parisi et al. 1999).

Retinal imaging has recently gained interest as a promising diagnostic tool for investigation of central nervous system diseases. It recalls Cicero's ‘The face is a picture of the mind as the eyes are its interpreter’ and demonstrates that the eye is the window to the brain. Optical coherence tomography (OCT) stands out from the available methods as it has been used for more than three decades (Petzold et al. 2017). It involves a noninvasive cross‐sectional imaging of the retina to assess changes in the retinal nerve fibre layer (RNFL) and, in more recent studies (Iorga et al. 2018; Pillay et al. 2018), changes in the ganglion cell layer. The decrease in the RNFL thickness correlates with degenerative processes (Park et al. 2014; Britze & Frederiksen 2018).

A more recent approach, namely retinal oximetry imaging, measures oxygen saturation of haemoglobin of the retinal blood vessels. The imaging procedure is noninvasive and reproducible with remarkably low variability in test–retest studies and in healthy cohorts (Palsson et al. 2012). Pathophysiological principles and novel biomarkers in several retinal diseases have been discovered, as well as possible applications for systemic and brain diseases (Stefánsson et al. 2019). Retinal oximetry has been used to asses retinal saturation in neurological disorders, such as Alzheimer's disease (Einarsdottir et al. 2016). Two recent studies have also demonstrated changes in retinal saturation in MS patients (Svrčinová et al. 2018; Einarsdottir et al. 2018). These studies have shown that the oxygen saturation is altered in MS patients. Einarsdottir et al. (2018) proved increased venous saturation (VS) and lower arterio‐venous (A‐V) difference, possibly due to atrophy and subsequent lower oxygen demand in MS patients after ON. Median time after onset of ON in that study was 2 years. In our previous study, we have reported changes in the early stage of ON, showing an increased A‐V difference in the affected eye, likely due to metabolic and inflammatory changes (Svrčinová et al. 2018). There are, to the best of our knowledge, no longitudinal studies of retinal saturation in MS patients.

We hypothesized that ON is associated with changes in the retinal blood oxygen saturation level and that these functional changes will correlate with subsequent structural changes in the RNFL thickness. Therefore, the main goal of this study was to assess changes in RNFL thickness and, simultaneously, in retinal oxygen saturation (arterial, venous and A‐V difference) in the affected and the unaffected eye and to evaluate the relationship between the two retinal imaging parameters.

## Materials and Methods

This was a prospective study following the tenets of the Declaration of Helsinki. There were enrolled 50 consecutive patients after giving their written informed consent, all Caucasian, 17 males. Inclusion criteria were as follows: acute (no more than 3 months) onset of ON as a symptom of MS with no previous steroid treatment of the current relapse. Patients with history of diabetes, glaucoma, refractive errors greater or equal ±6 dioptres, age‐related macular degeneration, cataract and patients after vitrectomy were excluded from the study group. The diagnosis of MS was confirmed based on the revised 2017 McDonald criteria (Thompson et al 2018). All participants underwent noninvasive spectrophotometric retinal oximetry and OCT at the baseline visit (V1) and after 6 months (V2). All participants were examined using Doppler ultrasound to exclude arterial stenosis and to asses bloodstream in ophthalmic artery.

### Optical coherence tomography

Optical coherence tomography (OCT) evaluations were performed using a Spectralis SD‐OCT (Heidelberg Engineering, Heidelberg, Germany). The device is able to record ocular movements via a confocal scanning laser ophthalmoscope (TrueTrac®; Heidelberg Engineering). The software adapts to ocular movements, allowing a correct examination. The RNFL examination was performed using a ring scan centred on the optic nerve head. The follow‐up study was performed using the automatic rescan mode. Before measurement, the pupil was dilated to a diameter of approximately 5 mm using 1% tropicamide eye drops (Mydriacyl; S.A. Alcon‐Couvreur N.V., Puurs, Belgium).

### Retinal oximetry

**Fig. 1:**
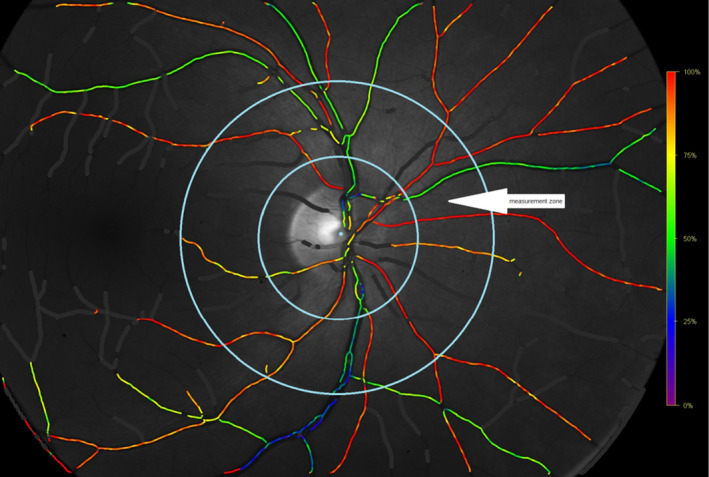
The measurement zone was marked by two circles concentric with the optic disc and with diameters of 1.5 times and three times the optic disc (measurement zone is marked with arrow). In the measurement zone, oxygen saturation was measured in all retinal arterioles and venules, with measuring restricted to above eight pixels of the vessel width.

An automated oximeter Oxymap T1 (Oxymap ehf., Reykjavik, Iceland) was used, and analyses were performed using the software oxymap analyzer (version 3.1.4; Oxymap ehf). Oxymap employs a fundus camera (Topcon DX‐50; Topcon inc., Tokyo, Japan) to measure haemoglobin‐bound oxygen in retinal vessels using the difference in light absorbance at 570 and 600 nm. Technical specifications of the device are described elsewhere (Geirsdottir et al 2012). Fundus photographs covering a 50° field were focused on the temporal edge of the optic disc, and the light flash was set at 50 Ws. Image analyses were performed following a Oxymap protocol for acquisition and analysis of Oxymap T1 oximetry images, version 21 November 2013, Oxymap ehf. In each image, the oxygen saturation was measured as described before (Svrčinová et al. 2018). Overall retinal oxygen saturation level was calculated according to Geirsdottir et al (2012). The A‐V difference was calculated as the difference between retinal arterial oxygen saturation and retinal venous oxygen saturation (Fig. 1) (Traustason et al. 2011).

### Neurosonology

The colour Doppler imaging was performed using 2–8 MHz linear probe (9L‐D, LOGIQ S8; GE Healthcare, Milwaukee, WI, USA). The lowest possible ultrasound intensity and the shortest possible sonication times were used to avoid any eye damage. We obtained peek systolic velocity (PSV) and end‐diastolic velocity (EDV) in the internal and external carotid artery on both sides and in the ophthalmic artery also bilaterally. These parameters were used to calculate the resistive index (RI) using the formula RI = (PSV − EDV)/PSV (Planiol 1973) and pulsatility index (PI) using the formula PI = (PSV − EDV)/*V*_mean_, where *V*_mean_ = 1/3 (PSV/EDV) + EDV (Gosling & King, 1974), to measure the degree of possible stenosis and alteration of blood flow that might have influenced results (Ferguson et al. 1999).

### Statistical analysis

All statistical analyses were conducted using custom scripts in matlab version R2018b, (MathWorks, Inc., Natick, MA, USA). Shapiro–Wilk's test of normality verified that most data did not have a normal distribution. Wilcoxon signed‐rank test was used for within‐subject pairwise comparisons of affected and unaffected eyes and differences between the visits. Correlations were evaluated using Spearman's correlation analysis. The value of p < 0.05 was adopted as the level of statistical significance.

## Results

At baseline visit (V1), arterial saturation (AS) was significantly increased in the affected eye compared to unaffected eye (median 99.5% versus 98.0%, p = 0.03, all pairwise comparisons performed using Wilcoxon signed‐rank test); at the 6‐month follow‐up visit (V2), there were no significant changes observed in oximetry (99.0% versus 99.0%, p = 0.29), see Table 1. There were no significant changes in VS in eye at either V1 (68.0% versus 66.0%, p = 0.87) and V2 (70.0% versus 68.0%, p = 0.24), see Table 1. A significant decrease in A‐V difference was detected in both eyes when compared V1 and V2: for affected eye (32.0% versus 29.0%, p = 0.004) and for fellow eye (31.4% versus 30.0%, p = 0.04), see Table 1. A modest increase in A‐V difference was detected in the affected eye compared to the unaffected eye at V1, but this did not reach significance (32.0% versus 31.4%, p = 0.09). During follow‐up, we did not observe any significant changes in retinal artery or vein diameter. There were no observed changes in blood flow in ophthalmic artery.

**Table 1:** Demographic data.

	All
*N* (CIS/RRMS)	50 (29/21)
Age [years median (range, IQR)]	37.0 (20.1–57.7, 13.6)
Gender (m/f)	32/18
EDSS at onset [median (range, IQR)]	1.5 (0.0–5.0, 1.0)
Duration of ON [months median (range, IQR)]	1 (0–3, 1)

Table shows descriptive statistics of the demographic data (number of subjects per diagnosis at Visit 1; age at Visit 1 in years; gender; Expanded Disability Status Scale (EDSS) score; and duration of optic neuritis (ON) at Visit 1 in months), including median, range, interquartile range (IQR).

CIS = clinically isolated syndrome, f = females, m = males, RRMS = relapsing–remitting multiple sclerosis.

**Fig. 2:**
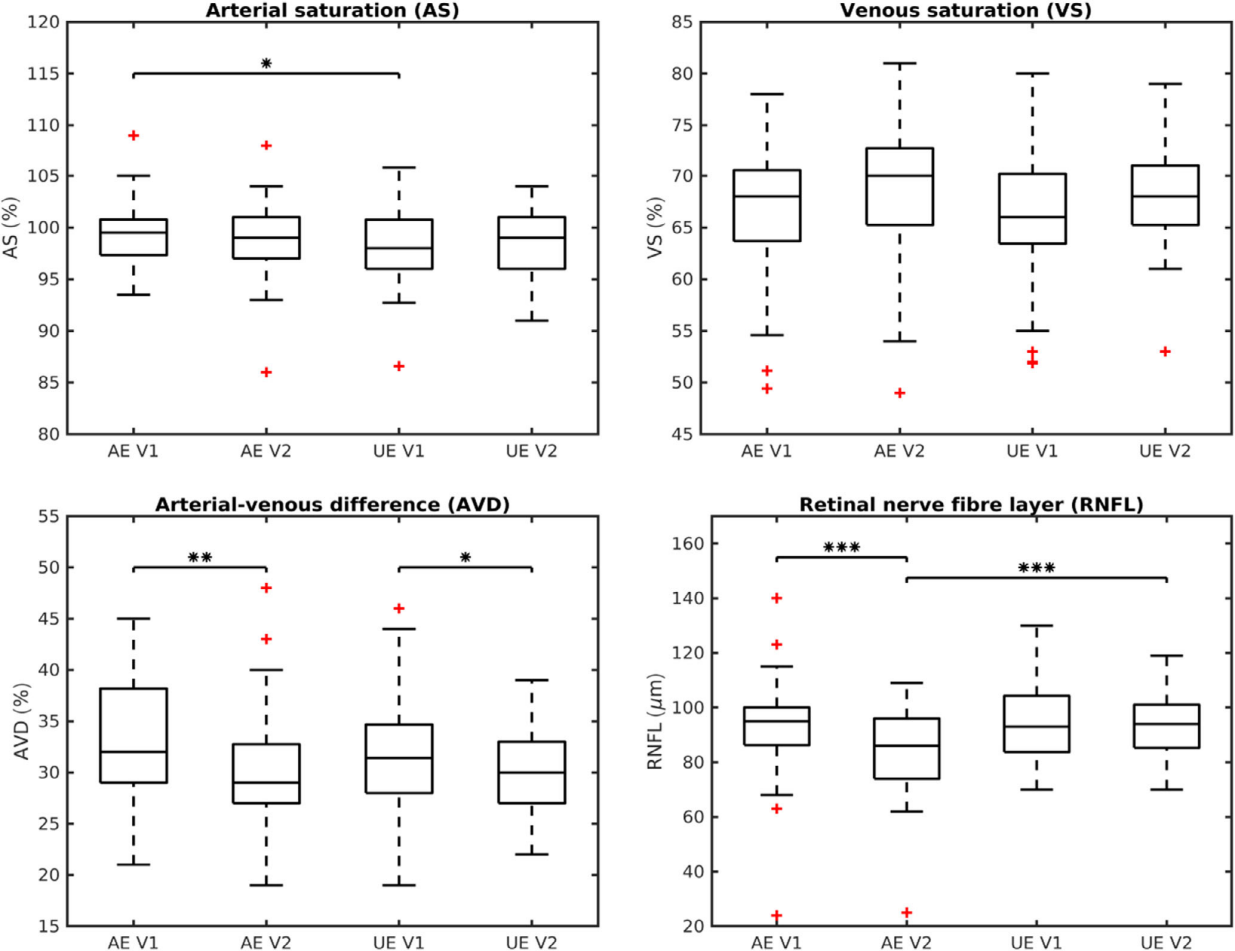
Summary box plots of changes in oximetry and optical coherence tomography values. It shows summary box plots of changes in retinal saturation values (AS – arterial saturation, VS – venous saturation, AVD – arterial‐venous difference) and the retinal nerve fibre layer (RNFL) in the affected eye (AE) and the unaffected eye (UE) at baseline visit (V1) and 6‐month follow‐up (V2). Thick horizontal lines indicate medians, boxes indicate interquartile range, whiskers indicate extreme values, and red plus signs stand for outliers. Asterisks and brackets indicate significant differences between variables or time‐points, * stands for p ≤ 0.05, ** for p ≤ 0.01. AS was significantly increased in the AE compared to UE at V1 (p = 0.03), AVD significantly decreased after 6 months in both eyes (for AE: p = 0.004; for UE: p = 0.04). Significant loss of RNFL was observed in the AE at V2, compared both to V1 (p = 0.0002) and to the UE (p = 0.0002).

At V1, there were no significant differences in the RNFL thickness measured by OCT (95 *μ*m versus 93 *μ*m, p = 0.69); however, significant loss of RNFL was confirmed in the affected eye at V2 (95 *μ*m versus 84 *μ*m, p = 0.0002), and this was also observed when compared to unaffected eye (86 *μ*m versus 94 *μ*m, p = 0.0002). See Table 1 and Fig. 2 for complete results.

**Fig. 3:**
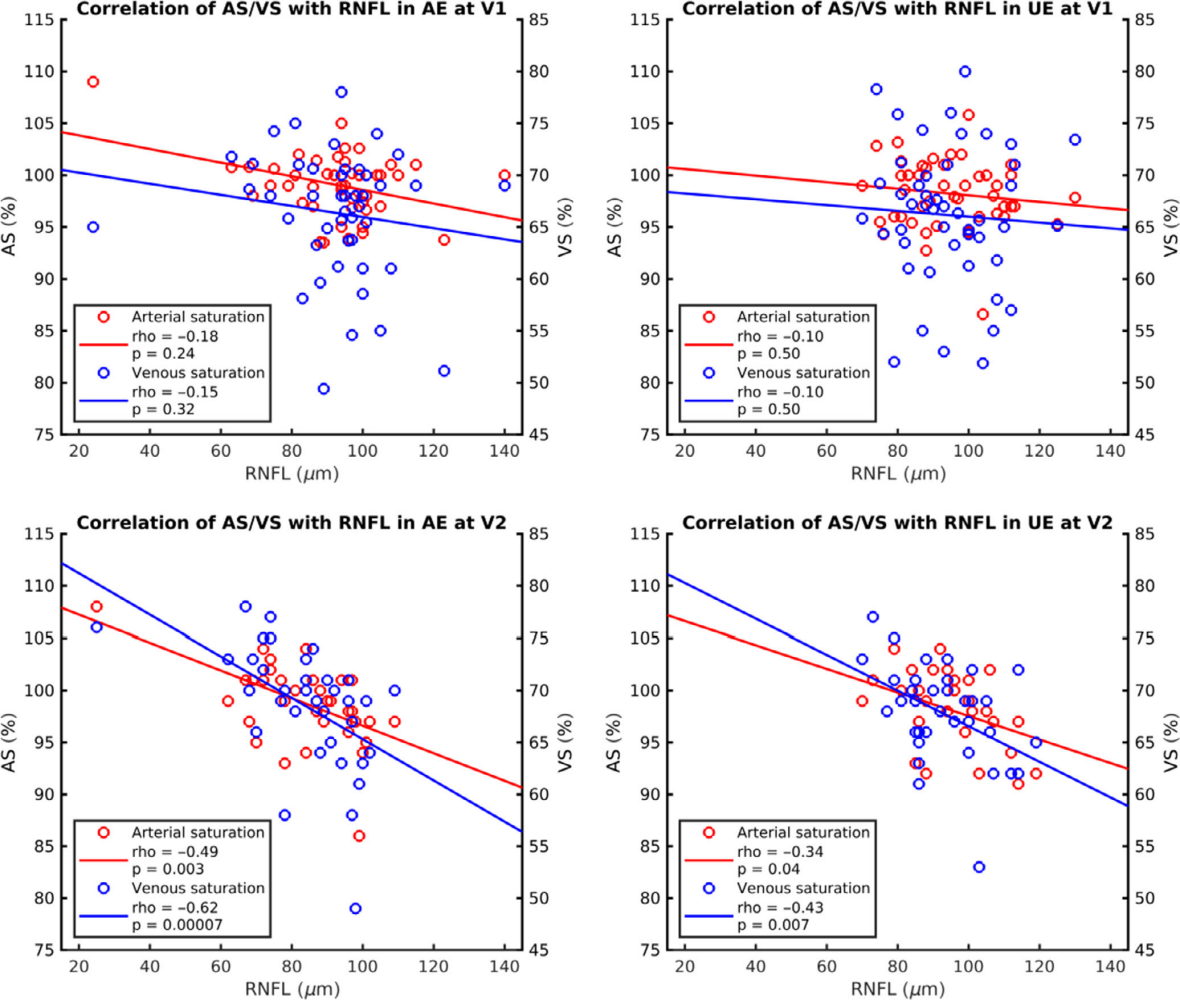
Correlation between oximetry and optical coherence tomography values. It shows scatter plots displaying correlation between saturation values and retinal nerve fibre layer (RNFL) thickness, and red colour represents AS and blue colour VS, remaining conventions see Fig. 1. No significant correlation was observed at V1, whereas a negative correlation between the RNFL and both AS (rho = −0.480, p < 0.05, Spearman's rank correlation coefficient) and VS (rho = −0.620, p < 0.05) emerged at V2.

There was no correlation between the RNFL and saturation values (neither AS nor VS) at V1, but at V2, there was a negative correlation between the RNFL and both AS (rho = −0.480, p = 0.003, Spearman's rank correlation coefficient) and VS (rho = −0.620, p = 0.00007). See Fig. 3.

## Discussion

To best of our knowledge, this is the first longitudinal study involving automatic retinal oximetry in brain disease. All previous studies in this field were cross‐sectional studies.

At baseline, we identified an increased level of AS and a trend towards increased mean A‐V difference in the affected eye as compared to the fellow eye. Interestingly, no such increase was found in VS.

The increase in AS is challenging to explain by physiological phenomena, and it could suggest some kind of unknown artificial mechanism in the imaging process since the quality of oximetry images did not differ. Similar increase of AS was observed in studies focusing on retinal vein occlusion where the explanation based on physiology was also difficult (Traustason et al. 2014; Jeppesen & Bek 2017; Osaka et al. 2019).

The findings of A‐V difference in retinal vessels in acute phase of the disease in our pilot study are not fully coherent in to our current study (Svrčinová et al. 2018). On the other hand in the pilot study, the A‐V difference reached the edge of statistical significance (p = 0.048) and in current study just slightly above the statistical significance (p = 0.09). Based on that data, almost the same trend of the A‐V difference increase could be expected in both studies. Based on the slight increase in the A‐V difference, it can be assumed that the change is only due to discrete (background) changes in metabolism. The modest increase in A‐V difference could be explained, in our opinion, by increased oxygen consumption in the affected eye during ON. First of all, greater metabolic turnover could be expected in ON because of the inflammation. The inflammatory process induces release of cytokines and other inflammatory mediators from activated peripheral T cells as they transfer the blood–brain barrier (Hoorbakht & Bagherkashi 2012). Burton et al. (2011) reported the retinal venous sheathing or inflammatory changes due to periphlebitis and suggested that vascular changes could be the initial event in ON due to MS because of the presence of vascular inflammation in the retina, a structure free of myelin and oligodendrocytes. On the other hand, there could be an alteration in the arterial flow, but the haemodynamic changes during acute ON are not clearly understood yet. In some studies, orbital blood flow velocities were decreased, whereas in others, they stayed unaffected or even increased (Akarasu et al. 2004; Hradilek et al. 2009; Karami et al. 2012). In this study, we found no changes in blood flow in the ophthalmic artery using Doppler imaging and no changes in retinal vessel diameter also. From these data, we could not expect changes in blood flow in central retinal artery and retina what bring us back for a role of increase oxygen consumption based on inflammatory changes in our opinion.

Apart from the observed differences at baseline, the main focus of this study was on the temporal development of saturation values. First, we observed no significant longitudinal changes in AS values or VS values alone. In contrast, A‐V difference was significantly reduced at the 6‐month follow‐up in both the affected and the unaffected eye, with more prominent changes being observed in the affected eye. This result indicates that there are changes in both eyes in terms of oxygen consumption during ON in MS. Decrease of the A‐V difference indicates decreased oxygen consumption or reduced oxygen uptake in the retina in MS patients even in silent phases of the disease. This finding is fully in agreement with a previous study by Einarsdottir et al. (2018) who found a decreased A‐V difference in chronic stage after ON (median time from ON was 2 years) compared to healthy controls. The most probable background for this is in our opinion neuronal atrophy (neurodegeneration). A similar mechanism of reduced oxygen uptake due to neuronal atrophy has been proposed in other neurological pathologies (Einarsdottir et al. 2016) or glaucoma (Olafsdottir et al. 2011).

Interestingly, the observed changes are only partly mirrored by the reduction of the RNFL thickness, which shows a different trend in each eye. There is a significant RNFL reduction in the eye with ON, but no such change in the fellow eye. The statistically significant reduction of the RNFL thickness after ON is consistent with previous reports (Petzold et al. 2017). This finding reveals interesting discrepancies between morphologic and metabolic sequelae of the disease in the retina. The A‐V difference reduction in the unaffected eye may reflect either systemic changes, possibly due to the MS treatment, or local metabolic abnormalities suggesting sub‐clinical inflammatory or neurodegenerative damage in the absence of overt clinical, electrophysiological or morphological signs of ON.

Another interesting point is the relationship between the retinal thickness and the oxygen saturation values in retinal vessels, which was first described by Mohan et al. (2016). They have found a correlation between saturation values (AS and VS) and the RNFL thickness and concluded that both physiological and artificial mechanisms could explain this phenomenon. In the presented results, the correlation between the RNFL and saturation values is only present in both eyes at V2, but not in the acute phase of the disease. This suggests that both eyes are affected in the acute phase from the metabolic point of view, and it highlights the value of oximetry compared to purely morphologic OCT.

The limitation of the study is that healthy controls were not included; in view of the results of our pilot study, where we observed large interindividual differences in the ophthalmic bloodstream, it seems more appropriate to compare the affected and unaffected eye of an each separately person where this interindividual variation does not affect the outcome.

## Conclusion

Based on our study, we claim that retinal oximetry is altered in both the affected and the unaffected eye in MS patients with unilateral ON. During the course of the disease, the retinal oxygen consumption decreases to a different degree in each eye and this change is not completely followed by changes in the RNFL thickness, suggesting either sub‐clinical ON or systemic effects in the clinically unaffected eye. It remains unclear whether the changes in ON are due to inflammation, neurodegeneration or combination of both. Since this is the first and initial longitudinal evaluation of the saturation changes in MS patients, the clinical value of these findings need to be deeper evaluated in the future studies.

**Table 2:** Oximetry and optical coherence tomography values.

	Median	Range	IQR	Median	Range	IQR	p
	AE			UE			AE versus UE
AS (%)							
V1	99.5	93.5–109.0	3.4	98.0	86.6–105.8	4.8	**0.03**
V2	99.0	86.0–108.0	4.0	99.0	91.0–104.0	5.0	0.29
p	0.56			0.95			
VS (%)							
V1	68.0	49.4–78.0	6.9	66.0	51.9–80.0	6.8	0.87
V2	70.0	49.0–81.0	7.5	68.0	53.0–79.0	5.8	0.24
p	0.05			0.08			
A‐V diff (%)							
V1	32.0	21.0–45.0	9.2	31.4	19.0–46.0	6.7	0.09
V2	29.0	19.0–48.0	5.8	30.0	22.0–39.0	6.0	0.37
p	**0.004**			**0.04**			
Artery thickness (px)							
V1	13.4	7.4–17.5	3.3	12.8	8.1–15.9	3.5	N/A
V2	13.2	8.3–17.2	3.2	12.5	7.8–17.1	2.7	N/A
p	0.23			0.99			
Vein thickness (px)							
V1	18.1	12.4–24.9	3.9	17.5	12.9–22.2	3.3	N/A
V2	18.2	12.9–22.5	4.8	17.9	12.9–23.6	3.8	N/A
p	0.30			0.44			
Image quality							
V1	8	6–10	2	8	7–10	1	0.87
V2	8	6–10	2	8	7–10	1	0.69
p	0.63			1.00			
RNFL (*µ*m)							
V1	95.0	24.0–140.0	13.8	93.0	70.0–130.0	20.5	0.69
V2	86.0	25.0–109.0	22.0	94.0	70.0–119.0	15.8	**0.0002**
p	**0.0002**			0.56			

Table shows descriptive statistics of the investigated parameters [retinal nerve fibre layer (RNFL) in *µ*m; retinal arterial saturation (AS) in %; retinal venous saturation (VS) in %; and arterio‐venous difference (A‐V difference) in %)] and quality assessment parameters [artery and vein thickness in pixels (px), retinal oximetry image quality on a scale 1–10 (1 is the worst, 10 is the best)], including median, range, interquartile range (IQR), number of patients with the available data and statistical test p values (Wilcoxon signed‐rank test), with significant values in bold font.

N/A = not available, V1 = baseline visit, V2 = follow‐up visit after 6 months.
